# The many faces of hematopoietic stem cell heterogeneity

**DOI:** 10.1242/dev.114231

**Published:** 2016-12-15

**Authors:** Mihaela Crisan, Elaine Dzierzak

**Affiliations:** 1 University of Edinburgh, BHF Centre for Cardiovascular Science, Scottish Centre for Regenerative Medicine, Edinburgh EH16 4UU, UK; 2 University of Edinburgh, Centre for Inflammation Research, Queens Medical Research Institute, Edinburgh EH16 4TJ, UK

**Keywords:** Hematopoiesis, HSC, Hematopoietic stem cells, Heterogeneity, Hematopoietic niche

## Abstract

Not all hematopoietic stem cells (HSCs) are alike. They differ in their physical characteristics such as cell cycle status and cell surface marker phenotype, they respond to different extrinsic signals, and they have different lineage outputs following transplantation. The growing body of evidence that supports heterogeneity within HSCs, which constitute the most robust cell fraction at the foundation of the adult hematopoietic system, is currently of great interest and raises questions as to why HSC subtypes exist, how they are generated and whether HSC heterogeneity affects leukemogenesis or treatment options. This Review provides a developmental overview of HSC subtypes during embryonic, fetal and adult stages of hematopoiesis and discusses the possible origins and consequences of HSC heterogeneity.

## Abstract (precis)

**Summary:** This Review takes a close look at hematopoietic stem cell heterogeneity during development and in the adult, and discusses several different ways in which this heterogeneity may arise.

## Introduction

**Fig. 1.**
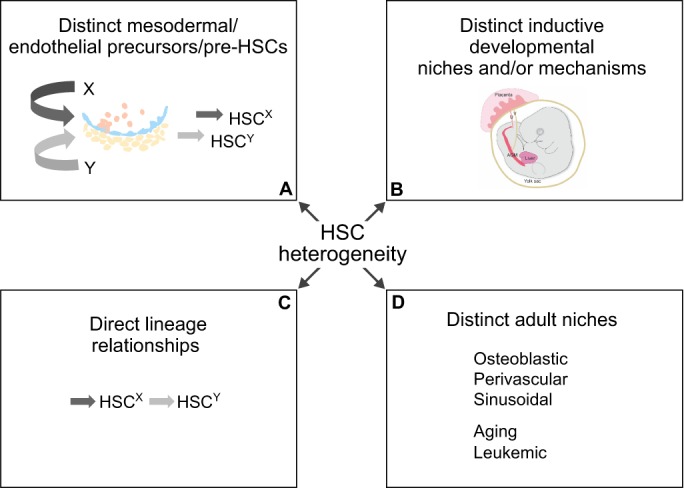
**Possible ways in which hematopoietic stem cell (HSC) heterogeneity may arise.** (A) HSC types may originate from distinct mesodermal cells that are prespecified early during development, distinct endothelial cells or from the maturation of pre-HSCs in the dorsal aorta. Hematopoietic cells are in pink, endothelial cells in blue and mesenchymal cells in yellow. (B) HSC types may be regulated by distinct inductive developmental tissues (for example the AGM, placenta, yolk sac, head) and may change as they migrate through the circulation; or by developmental niches, which may be vascular, hepatic, neural or bone; or by distinct developmental mechanisms (e.g. EHT). (C) HSC types may have direct lineage relationships whereby, after generation in the embryo, HSCs may upregulate or downregulate receptors allowing differential priming and responsiveness to specific signals that regulate lineage differentiation output, quiescence and/or other stem cell characteristics. (D) HSC types may be located in distinct adult bone marrow niches (for example osteoblastic, perivascular, sinusoidal or spleen) that may change qualitatively or quantitatively during development, aging and leukemia. It has yet to be determined which of these models is responsible for the generation of HSC heterogeneity, or indeed whether multiple models might work together. In addition, epigenetic modifications might also explain the presence of distinct HSC types. Understanding the origin of HSC heterogeneity will require further experimentation to identify unique markers of HSC subtypes, to better characterise different HSC niches and the localisation of HSC subtypes within them, and to precisely lineage trace HSC subtype generation throughout development.

Hematopoietic stem cells (HSCs) give rise to all cells in the blood lineage through the process of hematopoiesis. In order to do this, HSCs undergo self-renewal, retaining their multipotentiality and thus their function throughout life. While it is convenient to think of HSCs as a single, homogenous population, evidence from recent years does not support this view. The concept that HSCs can be sorted to homogeneity and that each HSC behaves identically in reconstitution assays is under challenge. Clonal HSC transplantations and refined cell sorting have identified HSC subtypes with different functional properties, including differences in repopulation kinetics (Benz et al., 2012; Dykstra et al., 2007; Muller-Sieburg et al., 2012; Sieburg et al., 2006; Verovskaya et al., 2013), cell cycle status (Wilson et al., 2008), self-renewal abilities (Ema et al., 2014, 2005) and multilineage differentiation output (Benz et al., 2012; Dykstra et al., 2007; Muller-Sieburg et al., 2012; Sieburg et al., 2006; Verovskaya et al., 2013). Although the evidence for HSC heterogeneity is strong, its source has yet to be identified (see Fig. 1). Are HSC subtypes programmed intrinsically during development? Or, do all HSCs begin life identically, with heterogeneity determined by extrinsic factors encountered during the migration and colonisation of HSCs in different developmental tissues and their respective niches? These important issues, as well as the physiological relevance of HSC heterogeneity, are beginning to be addressed. In this Review, we summarise the current knowledge regarding HSC heterogeneity in the mouse, as well as recent efforts to tease apart different HSC subtypes and how they might arise. Different HSC subtypes may be defined using refined cell sorting techniques, based on either HSC cell surface markers, endogenous reporters or differing responsiveness to signalling pathways. We discuss these different phenotypic classifications of HSC subtypes and consider the developmental aspects of HSC emergence that might give rise to such heterogeneity.

## HSC heterogeneity in adult bone marrow

The Muller-Sieberg group was among the first to define HSC heterogeneity by observing the repopulation kinetics of single HSCs following transplantation into irradiated or cKit receptor tyrosine mutant (*W/W*) recipient mice (Sieburg et al., 2006). Statistical analyses showed 16 types of kinetic repopulation patterns measured over 8 months in recipients that received a clonal stem cell transplantation from whole bone marrow. Clonal transplantation of sorted Lin^−^ Rho^−^ side population (SP; see below) HSCs showed selective enrichment of only some kinetic subsets of all of the HSC types found in bone marrow. Moreover, daughter HSCs from primary hosts, when transplanted into secondary hosts, followed the same repopulation kinetics as the original transplanted HSC (Muller-Sieburg et al., 2002). This result predicted that distinct stable HSC subtypes exist and that HSC heterogeneity can be stably propagated.

Heterogeneity in the ability of HSCs to self-renew has been examined in multiple serial transplantation experiments by measuring the lifetime of HSC clones derived from whole bone marrow between 4 months and 2 years post-transplantation (Muller-Sieburg et al., 2002). The conclusion was that self-renewal did not contribute to the heterogeneity of the adult HSC compartment, and that HSCs may have an intrinsic pre-determinate fate. Self-renewal capacity was also quantified in single sorted (CD34^−^ Lin^−^ cKit^+^ Sca1^+^) HSCs by competitive repopulation assays in both primary and secondary recipients, and the results showed that great diversity exists in the repopulating activity between HSC clones (Ema et al., 2005). Although all myeloid and lymphoid (B and T cell) lineages were reconstituted from single HSC clones, the level of reconstitution in each of the lineages was different.

As well as the ability to self-renew, the intrinsic cell cycle activity of individual HSCs may contribute to HSC heterogeneity. The results of two label-retention studies in mice – BrdU labelling or histone marking with H2B-GFP synthesized during the active cell cycle upon recombination in SCL-tTA transgenic mice – show that most HSCs are dormant, label-retaining cells (Wilson et al., 2008). Dormant HSCs can reversibly become active upon stimulation with granulocyte colony stimulating factor (G-CSF; also known as Csf3) or in response to bone marrow injury. BrdU label-retaining cell data were further incorporated in computational models to enable an estimation of the dormant and the active CD34^−^ Lin^−^ cKit^+^ Sca1^+^ CD150^+^ CD48^−^ HSC turnover rates (van der Wath et al., 2009; Wilson et al., 2009). The active HSCs are responsible for the regular, everyday maintenance of the hematopoietic system, while the dormant HSCs divide rarely but can be activated upon injury. The analysis predicted that dormant HSCs cycle once every 149-193 days, whereas active HSCs cycle once every 28-36 days (van der Wath et al., 2009; Wilson et al., 2009).

## Multilineage hematopoietic outputs characterise HSC subtypes in the bone marrow

**Fig. 2.**
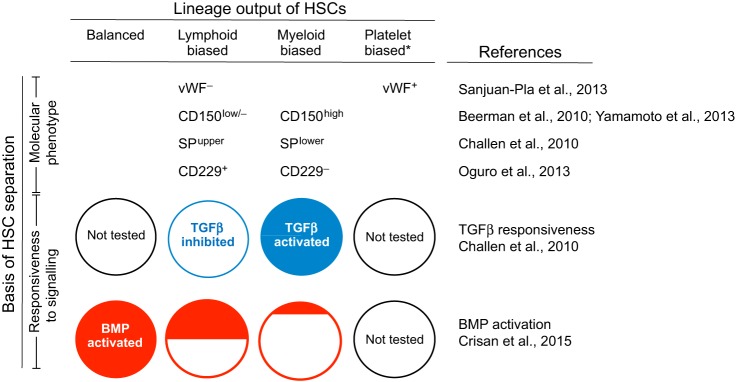
**The relationship between bone marrow HSC lineage output, phenotype and responsiveness to signalling pathways.** CD229, CD150, vWF and Hoechst dye efflux (SP) were used together with canonical HSC markers (Lin^−^ Sca1^+^ cKit^+^ or Lin^−^ Sca1^+^ cKit^+^ CD48^−^ CD150^+^) to enrich myeloid-biased, lymphoid-biased or platelet-biased HSCs (*here, platelet-biased HSCs also include platelet-myeloid-biased HSCs). In addition to the canonical HSC markers used, HSC fractions sorted by Yamamoto et al. (2013) were CD34^−^. Myeloid-biased HSCs are generally activated by the TGFβ signalling pathway (blue), whereas lymphoid-biased HSCs are inhibited (white). All myeloid-lymphoid (balanced) HSCs are BMP-activated (red). The myeloid-biased HSCs are mostly non-BMP-activated (white). The lymphoid-biased HSCs are equally distributed between BMP-activated and non-BMP-activated fractions.

The primary function of an HSC when transplanted into an irradiated/myelodefective adult mouse recipient is the production of mature hematopoietic cells that include cells of the myeloid (granulocyte, macrophage), erythroid, platelet/megakaryocyte and lymphoid (B and T cell) lineages. Measurement of mature hematopoietic cell lineage outputs in the peripheral blood at 4-6 months following clonal stem cell transplantation revealed that HSCs fall into several subtypes. These could be retrospectively classified as being ʻmyeloid-biased' (also called ʻlymphoid-deficient'), ʻlymphoid-biased' (also called ʻmyeloid-deficient') and ʻmyeloid-lymphoid balanced', based on the measurement of the predominant lineages within the total output of donor-derived HSCs (Cho et al., 2008; Muller-Sieburg et al., 2012) or based on the relative contribution of each donor-derived lineage to the total myeloid plus lymphoid cell population (both donor-derived and recipient) (Dykstra et al., 2007) (Fig. 2). The HSC subtype with a myeloid-lymphoid balanced output predominates in young adult bone marrow from C57BL/6 mice (Dykstra et al., 2007). However, the distribution of the HSC subtypes changed between young and old (>38 week) mice, with an increase in the representation of the myeloid-biased HSC subtype in older mice (Cho et al., 2008; Dykstra et al., 2007). Secondary and tertiary transplantations showed that the balanced and myeloid-biased HSCs are stable subtypes, yielding the same lineage outputs in their daughter HSCs over long periods of time (Benz et al., 2012; Cho et al., 2008; Dykstra et al., 2007; Muller-Sieburg et al., 2002).

Recently, other HSC subtypes have been described. ʻPlatelet-biased' and ʻplatelet-myeloid-biased' HSCs were identified through the transplantation of bone marrow-derived HSCs either expressing or not expressing von Willebrand factor (vWF) (Sanjuan-Pla et al., 2013). Platelet-biased and platelet-myeloid-biased HSCs were enriched in the vWF^+^ subset, and vWF^+^ HSCs showed higher myeloid and lower lymphoid contributions compared with vWF^−^ HSCs (Fig. 2).

Others have classified HSC subtypes into long-, intermediate- and short-term (LT, IT and ST) repopulating HSCs based on the reconstitution kinetics over a period of 24 weeks following clonal HSC transplantation into primary recipients and 20 weeks in secondary recipients (Yamamoto et al., 2013). Although all HSCs were multilineage repopulating, the degrees of donor chimerism in the myeloid lineage compared with the lymphoid lineage varied. LT-HSCs exhibited high donor chimerism in all five lineages tested – neutrophil/monocyte, erythrocyte, platelet, B cell and T cell – that reached a threshold at 4 weeks post-transplantation and maintained this threshold at 24 weeks in the primary recipient and also after secondary transplantation. IT-HSCs displayed lower levels of chimerism over the same period but began to lose myeloid and erythroid output upon secondary transplantation. The ST-HSCs do not provide a persistent level of chimerism in any of the five lineages in the primary recipient over the 24-week period, and secondary transplantations showed a predominantly lymphoid output.

The relationship between the LT/IT/ST classification system and the other HSC subtype classifications (Cho et al., 2008; Dykstra et al., 2007; Yamamoto et al., 2013) was assessed in a recent review (Ema et al., 2014). Comparisons of the data showed that most of the LT-HSCs in bone marrow were myeloid-biased, the majority of the ST-HSCs were lymphoid-biased, and that the IT-HSC subtype contained a mixed (balanced and lineage-biased) population of HSCs. Since active HSCs enter the cell cycle every month and the dormant HSCs every 5 months (Wilson et al., 2008), it would be interesting to know whether the HSC subtypes based on cell cycle and self-renewal activity overlap with the HSC subtypes based on lineage output and long-term persistence in serial transplantations.

## Molecular properties associated with bone marrow-derived HSC subtypes

Evidence from a growing number of studies suggests that the characteristic properties of HSC subtypes are primed intrinsically. However, it has been difficult to obtain a molecular signature of the different HSC subtypes associated with these properties because of their inability to be prospectively isolated. HSCs are typically purified from the adult bone marrow based on a combination of markers, such as being Lin^−^ Sca1^+^ cKit^+^ CD150^+^ CD48^−^ (Kiel et al., 2005). Refinements in this isolation procedure revealed that the levels of CD150 (Slamf1), cKit and Sca1 (Ly6a) expressed on the surface of HSCs differ, and that these differences are associated with HSC functional heterogeneity when tested in repopulation assays (Kent et al., 2009; van der Wath et al., 2009; Wilson et al., 2009; Morita et al., 2010). Indeed, compared with the CD150^medium^ and CD150^low^ HSCs, CD150^high^ HSCs show greater self-renewal capacity in serial transplantation assays. Moreover, flow cytometric index sorting combined with single-cell RNAseq and single-cell transplantation verify that, based on the CD150 and Sca1 expression levels, HSCs differ in the kinetics of cell division and differentiation. CD150^+^ Sca1^low^ cells were significantly more proliferative than CD150^+^ Sca1^high^ cells, suggesting that the latter are the dormant HSCs. In combination with index sorting data, single-cell gene expression signatures suggest that dormant HSCs have the ability to respond to stress and injury. Furthermore, overlay between single-cell gene expression data and single-cell transplantation results uncovered a difference between repopulating and non-repopulating HSCs based on homing abilities.

cKit is another molecular marker that has been used to distinguish between HSCs with different repopulation characteristics following transplantation. Purified HSCs that express intermediate levels (cKit^int^), which are normally quiescent *in situ* in the steady-state bone marrow, are highly proliferative after transplantation and can efficiently repopulate secondary and tertiary recipients. By contrast, cKit^high^ HSCs have low expansion capacity and reduced repopulating activity in primary recipients and after serial transplantations (Grinenko et al., 2014). These findings were supported by cell cycle analysis, which showed that cKit^int^ HSCs are quiescent compared with the cycling cKit^high^ HSCs. Transcriptomic analyses show molecular differences between these two HSC subtypes: genes related to cell adhesion and VEGFR signalling were upregulated in cKit^int^ HSCs compared with cKit^high^ HSCs, whereas cell cycle genes were downregulated in cKit^int^ HSCs compared with cKit^high^ HSCs. The existence of two HSC subtypes based on cKit expression was also demonstrated by Shin et al. (2014). In this study, purified cKit^low^ HSCs exhibited long-term reconstitution potential and enhanced self-renewal capacity when transplanted into primary and secondary recipients, in contrast to cKit^high^ transplanted HSCs. Both subpopulations reconstitute irradiated recipients; however, the ability of the cKit^high^ population to self-renew was lost 4 weeks after the secondary recipients were transplanted (Grinenko et al., 2014). Together, these two studies demonstrate both *in vivo* and *in vitro* that different HSC subtypes marked by varying levels of cKit are hierarchically organised, and that an increasing level of cKit expression corresponds with the start of differentiation. Thus, distinct levels of cKit expression are associated with specific functional repopulation and self-renewal characteristics of HSC subtypes.

HSCs that express different levels of CD150 and cKit have also been examined for their association with hematopoietic lineage output following transplantation (Fig. 2). In one study it was shown that differing levels of CD150 expression distinguish HSCs with different lineage outputs (Beerman et al., 2010). Upon transplantation of 10 or 180 sorted HSCs per recipient mouse in competitive repopulation assays, CD150^high^ HSCs gave a predominant myeloid-biased output, whereas CD150^low^ gave a lymphoid-biased lineage output. Interestingly, when two HSC populations defined by the cKit surface expression level were examined by FACS for CD150 expression, no differences in the level of CD150 were found. Moreover, cKit^high^ and cKit^int^ HSCs showed comparable lineage outputs as measured in the peripheral blood of primary recipients upon transplantation in limiting dilution experiments (Shin et al., 2014). In the same study, however, *in vitro* assays demonstrated that cKit^high^ HSCs exhibit a megakaryocytic differentiation bias.

Hoechst dye efflux is another method of HSC isolation and produces a population termed the side population (SP) (Goodell et al., 1996). Different SP subfractions correlate with HSC subtypes. For example, the lineage output of clonally transplanted Lin^−^ Sca1^+^ cKit^+^ bone marrow cells from the lower SP region was enriched in myeloid-biased HSCs, whereas that from the upper SP region was enriched in lymphoid-biased HSCs (Challen et al., 2010). In addition, the CD229 (Ly9) marker was used to further isolate HSCs within the Lin^−^ Sca1^+^ cKit^+^ CD150^+^ CD48^−^ CD244^−^ bone marrow fraction. CD229^−^ cells contained 79% myeloid-biased HSCs, 7% balanced and 14% lymphoid-biased HSCs. CD229^+^ cells contained 22% myeloid-biased, 22% balanced and 56% lymphoid-biased HSCs (Oguro et al., 2013). Hence, high-purity sorting of HSCs based on cell surface markers as well as SP regions indicate a correlation between molecular phenotype and lineage output.

It was previously suggested that adult bone marrow myeloid-biased or lymphoid-biased HSC subtypes could be distinguished by their responsiveness to factors released by their surrounding microenvironment. For example, the loss of responsiveness of the myeloid-biased HSCs to interleukin 7 (IL7) may be due to the downregulation of IL7 receptor (IL7R) (Muller-Sieburg et al., 2004). Lymphocytes derived from myeloid-biased HSCs showed downregulation of IL7Rα gene and protein expression as compared with those derived from lymphoid-myeloid balanced HSCs. Indeed, another study reported that lymphoid-myeloid balanced HSCs show significantly higher expression of lymphoid gene regulators, such as *Pax5*, *Il7r*, *E2a* (*Tcf3*) and *Ikaros* (*Ikzf1*), than myeloid-biased HSCs (Benz et al., 2012).

Interestingly, myeloid-biased and lymphoid-biased HSCs are differentially responsive to the TGFβ signalling pathway upon exposure *in vitro* or injection of TGFβ1 into mice *in vivo* (Fig. 2)*.* In all cases, TGFβ promotes proliferation and myeloid differentiation in a dose-dependent manner, specifically in myeloid-biased HSCs (Challen et al., 2010). It induces opposing transcriptional responses in the two HSC fractions for genes related to cell cycle activation, lymphoid versus myeloid differentiation genes and even oncogenes. Recently, activation of BMP signalling in the bone marrow was found to be associated with balanced and lymphoid-biased HSCs, whereas more myeloid-biased HSCs were found in the non-BMP-activated fraction (Crisan et al., 2015) (Fig. 2). Transcriptomic data from these sorted HSC fractions showed that gene targets of decitabine, a small molecule hypomethylating agent that inhibits DNA methyltransferase (Kantarjian et al., 2006), were significantly upregulated in BMP-activated HSCs and significantly downregulated in non-BMP-activated HSCs. Decitabine is used today to treat patients with myelodysplastic syndrome and acute myeloid leukemia (Kantarjian et al., 2003).

Altogether, these studies have yielded insight into the some of the intrinsic molecular differences between HSC subtypes.

## Exploring the possible origins of HSC heterogeneity

Given the relationship between HSC lineage output, molecular phenotype and responsiveness to signalling pathways, it is of great interest to understand the origins of HSC heterogeneity. There are several different scenarios that might explain how HSC heterogeneity arises, including the influence of distinct HSC niches in the adult bone marrow, a direct lineage progression from one HSC type to another, distinct embryonic origins, and/or different developmental microenvironments (Fig. 1). For the remainder of this Review, we discuss each of these scenarios and evaluate the evidence both for and against each one.

### The bone marrow microenvironment and HSC heterogeneity

Adult HSC function may be related to HSC localisation within the bone marrow niches. Recent studies focusing on the mouse bone marrow niche have demonstrated that the HSC-supportive niches are composed of combinations of highly diverse ‘stromal’ cell types. Cells that comprise these niches include osteoblasts, macrophages, megakaryocytes, sympathetic nervous system cells, endothelial cells, and perivascular and perisinusoidal mesenchymal stromal/stem cells (Boulais and Frenette, 2015). This implies that distinct stromal cell factor combinations and/or distinct cell contacts between different types of stromal cells and HSCs can control HSC quiescence, survival, proliferation, self-renewal and mobilisation/or retention in their niche (Wohrer et al., 2014), and hence could form the basis of what is described as HSC heterogeneity.

Some bone marrow HSCs are in direct contact with osteoblasts. Conditional ablation of osteoblasts results in bone loss and significantly decreases the number of HSCs as well as myeloid, erythroid and lymphoid progenitors (Visnjic et al., 2004). This is in line with a converse study that showed an increase in the number of HSCs concomitant with an increased number of osteoblasts as a result of the osteoblast-specific conditional inactivation of *Bmpr1a* (Zhang et al., 2003). These results demonstrate that HSC numbers are dependent on niche size, which is mediated by the BMP signalling pathway. However, bone marrow HSCs are also found in contact with macrophages, megakaryocytes, endothelial cells and perivascular cells. Bone marrow macrophages release CXCL12 (SDF1), which is a potent chemoattractor of HSCs, and osteocalcin (Bglap), and both act to support osteoblast survival, thereby retaining HSCs in their niche (Chow et al., 2011; Christopher et al., 2011). Deletion of CD169 (Siglec1)^+^ macrophages leads to decreased retention of HSCs in the mesenchymal niche in the bone marrow and consequently HSCs are mobilised in the blood stream (Chow et al., 2011). In line with this study, G-CSFR (Csf3r) has been reported to signal in monocytes to mobilise HSCs in the blood stream by suppressing the supportive role of osteoblasts and disrupting the CXCR4/CXCL12 axis (Christopher et al., 2011). Owing to the intimate cross-talk between osteoblasts and macrophages in their regulation of HSC maintenance, it is difficult to dissociate these cell types as being distinct HSC-supportive niches. In contrast to macrophages, the sympathetic nervous system facilitates HSC mobilisation and migration (Chow et al., 2011; Katayama et al., 2006).

HSCs are also found in direct contact with megakaryocytes, which are cells at the crossroads of regulating HSC quiescence and expansion. Megakaryocytes normally secrete cell cycle regulators such as thrombopoietin, TGFβ1 and CXCL4, which keep HSCs in G0 of the cell cycle (Nakamura-Ishizu et al., 2014; Bruns et al., 2014; Zhao et al., 2014). However, under the chemotherapeutic stress of the small molecule 5-flurouracil, megakaryocytes secrete FGF1 and downregulate TGFβ1, stimulating the expansion of HSCs (Zhao et al., 2014).

Endothelial cells are essential for the self-renewal and repopulation activity of HSCs through release of angiocrine factors that activate the Notch or Akt signalling pathways (Butler et al., 2010; Kobayashi et al., 2010; Poulos et al., 2013). Recent work demonstrated that more than 94% of HoxB5-marked HSCs in the bone marrow are found in an ablumenal position, directly attached to VE-cadherin (Cdh5)-expressing endothelial cells (Chen et al., 2016). These HoxB5-marked HSCs represent between 7% and 35% of phenotypic HSCs when classic markers are used – for instance Lin^−^ Sca1^+^ cKit^+^ CD150^+^ CD48^−^ – suggesting that this compartment remains heterogeneous.

Other recent studies have shown that bone marrow HSCs are in contact with perivascular mesenchymal cells, which regulate HSC cell cycle activity by secreting stem cell factor (SCF; also known as Kitl) and CXCL12 (Ding and Morrison, 2013; Ding et al., 2012; Omatsu et al., 2010). Conditional deletion of *Scf* in endothelial cells and Leptin receptor-expressing perivascular cells results in decreased bone marrow HSC numbers (Ding et al., 2012). Deletion of *Scf* from hematopoietic cells, osteoblasts and nestin-expressing cells did not affect HSC number or function. When CXCL12-abundant reticular (CAR) perivascular cells were depleted, HSCs were also reduced in number and were more quiescent, suggesting a key role for these cells and the chemokine that they secrete in controlling HSC proliferation (Omatsu et al., 2010). Whether CXCL12-expressing perivascular cells are mainly found in the arteriolar wall or around sinusoids remains unclear. It was shown that arteriolar perivascular cells expressing NG2 (Cspg4) maintain HSC quiescence (Kunisaki et al., 2013). However, deep imaging of bone marrow shows that the non-dividing HSCs are mainly associated with sinusoidal perivascular Leptin receptor-expressing cells (Acar et al., 2015). HSC cell cycle regulation is thus based on their proximity to one or other stromal cell type, which cooperate with endothelial cells to support HSCs (Greenbaum et al., 2013).

Finally, HSC localisation in the bone marrow niche can be modulated intrinsically by nuclear factor-like 2 (Nrf2; also known as Nfe2l2), a master regulator of the oxidative stress response. This factor, expressed by HSCs themselves, acts as a negative regulator of cell cycle progression by partially regulating CXCR4 expression (Tsai et al., 2013).

On their own, local blood perfusion and hypoxia can functionally separate HSC populations in the bone marrow niche (Lévesque and Winkler, 2011).

Altogether, these observations suggest that HSC heterogeneity is supported by the high diversity of cell types found in the supportive niches of the bone marrow; however, it remains unknown whether HSC heterogeneity is unique to the bone marrow or whether some HSC subtypes are developmentally determined.

### The developmental counterparts of adult HSC subtypes

**Fig. 3.**
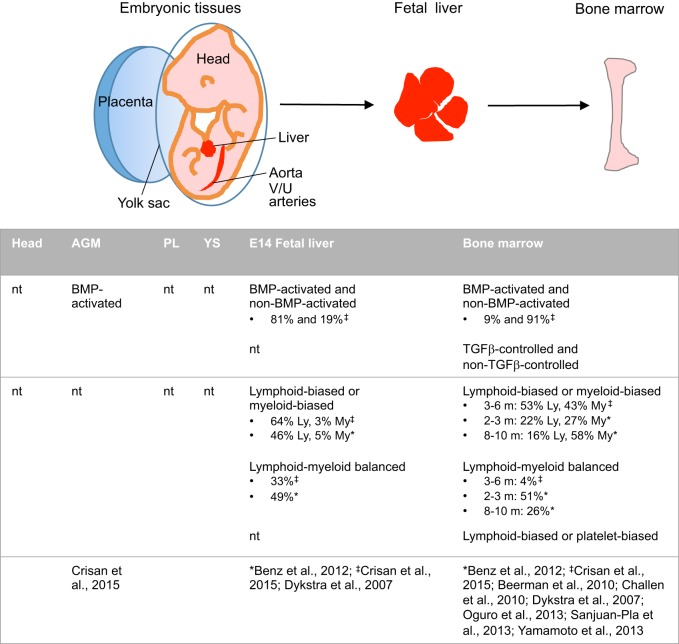
**Distribution of HSCs and heterogeneity in developmental niches.** HSC development is time and tissue dependent. HSCs are located in multiple intraembryonic tissues, including the aorta, vitelline and umbilical arteries (V/U), head and liver, and the extraembryonic tissues, yolk sac (YS) and placenta (PL). At the fetal stage, the liver provides niches for HSC expansion and maintenance. In the adult, HSCs are found in a variety of niches in the bone marrow. HSC heterogeneity is summarised beneath in terms of types (top) and subtypes (middle), with associated references. Only percentages comparing fetal liver and bone marrow are shown. nt, not tested; Ly, lymphoid-biased; My, myeloid-biased. Bone marrow values refer to 3-6, 2-3 or 8-10 months (m) old.

During development of the adult hematopoietic system, HSCs are localised in several different microenvironments that are not only supportive but also elicit unique inductive and expansion properties (Dzierzak and Speck, 2008) (Fig. 3). Initially, within the inductive microenvironment of the embryo, HSCs arise from specialised endothelial cells in the aorta and other major vasculature (de Bruijn et al., 2002, 2000) that undergo transdifferentiation to an HSC fate. HSCs can also be detected in the yolk sac (Kumaravelu et al., 2002), placenta (Gekas et al., 2005; Ottersbach and Dzierzak, 2005) and head of the embryo (Dzierzak and Speck, 2008; Li et al., 2012). As shown in the mouse model, these HSCs are robust, self-renewing and can achieve high-level, long-term multilineage engraftment into adult irradiated recipients following clonal transplantation (Taoudi et al., 2008). Following their generation and short-term maintenance in the vascular regions of the embryo, HSCs migrate and colonise the fetal liver, where they expand and are maintained until shortly before birth, at which point they again migrate and finally reside in the bone marrow in HSC-supportive niches (Dzierzak and Speck, 2008) (Fig. 3). Thus, HSCs experience several distinct microenvironments during development, and accumulating evidence suggests that HSC heterogeneity begins at these early developmental stages.

HSCs of the embryo, fetal liver, neonatal bone marrow and adult bone marrow have been studied and compared for properties such cell cycle, self-renewal and lineage output. Almost 100% of HSCs in the E14.5 fetal liver were found to be actively cycling, compared with only ∼10% of adult bone marrow HSCs – indeed, most adult bone marrow HSCs are quiescent (Fleming et al., 1993; Morrison et al., 1995; Bowie et al., 2006). Little is known regarding the cell cycle status of HSCs during the inductive phase in the embryo, since the paucity of aortic HSCs precludes precise measurements. Only very rare hematopoietic cells expressing the proliferation marker Ki67 are detected on E11.5 aorta-gonad-mesonephros (AGM) frozen sections (Mirshekar-Syahkal et al., 2013). The E10 mouse aortic region was also examined in thick slices stained for CD31 (Pecam1), cKit and phospho-histone H3.3 (PHH3), and showed a low mitotic index for the hematopoietic cluster cells (Boisset et al., 2015). Differences have also been found in the self-renewal properties of HSCs over the developmental timecourse. Fetal liver and umbilical cord HSCs appear to have more proliferative potential and can be serially transplanted more times that adult bone marrow HSCs (Bowie et al., 2007; Harrison et al., 1997), suggesting that developmentally young (fetal and neonatal) HSCs are more robustly self-renewing. The higher proliferation ratio in the fetal liver compared with the adult bone marrow was also observed more recently, and the authors suggested that it was driven by a higher mitochondrial content and activity, coupled with elevated oxygen consumption, in the HSCs purified from fetal liver (Manesia et al., 2015). However, the existence and possible influence of distinct niches – and the distinct cellular types within them – cannot be ruled out. For example, only ∼45% of HSCs were lost in E14.5 fetal liver in which perivascular (NG2-expressing) cells were deleted as compared with control wild-type fetal liver cells (Khan et al., 2016), suggesting that other external stimuli or cells control HSC expansion and/or maintenance. It would be interesting to investigate whether a certain HSC subtype is associated with portal vessels containing NG2-expressing perivascular cells.

As early as the fetal liver stage, HSC subtypes can be found that show heterogeneity in their lineage output. Clonal transplantations of mouse fetal liver HSCs show HSC subtypes similar to those found in the adult bone marrow: lymphoid-deficient balanced and myeloid-deficient were found at E14.5 (Benz et al., 2012). Interestingly, the proportions of these HSC subtypes are reversed in the fetal liver as compared with their adult bone marrow counterparts. Whereas with aging, the adult bone marrow contains proportionally more myeloid-biased (or lymphoid-deficient) HSCs (Benz et al., 2012; Cho et al., 2008), the E14 fetal liver contains 10-fold more myeloid-lymphoid balanced than myeloid-biased HSCs (Benz et al., 2012). The proportional representation of HSC subtypes might be dependent to some degree on the different microenvironments of the fetal and/or adult stages as a consequence of different extrinsic locally released microenvironmental factors. For example, the bone marrow niche might produce more factors that maintain myeloid-biased HSCs, whereas the fetal liver might be rich in factors that expand/maintain myeloid-lymphoid (balanced) HSCs.

### Molecular changes during HSC emergence: fetal liver compared with bone marrow

The intrinsic gene regulatory programmes that dictate the bifurcation of HSCs into subtypes is of interest, since this may affect reprogramming strategies to direct non-hematopoietic cells to HSC fate. Molecular studies of HSCs isolated from different developmental stages have highlighted some genes that may play a role in HSC subtype appearance and/or behaviour. In two separate studies, gene expression profiles of fetal liver-derived versus adult bone marrow-derived HSCs revealed that the Sox17 transcription factor is specifically expressed in fetal and neonatal (up to 4 weeks) HSCs, but not in adult HSCs (He et al., 2011; Kim et al., 2007). Sox17 is expressed in the hemogenic endothelium, emerging HSCs and also in the intra-aortic cell clusters (Clarke et al., 2013; Nobuhisa et al., 2014). Germline deletion of *Sox17* causes severe fetal hematopoietic defects and an absence of definitive HSCs. Conditional deletion of *Sox17* during mid-gestation using endothelial-directed Cre [driven by *Tie2* (*Tek*) or VE-cadherin] resulted in lethality at E13.5. When Sox17 was deleted in hematopoietic cells in 2- to 6-day-old neonates using *Mx1-Cre*, all mice died by 14 days after birth. However, conditional deletion of *Sox17* in hematopoietic cells of 6-week-old mice using *Mx1-Cre* did not affect hematopoiesis. These data suggest that Sox17 is required for the maintenance of neonatal but not adult HSCs, and that Sox17 is a key determinant of the earliest stages of HSC identity. Additional members of the SOX family, *Sox7* and *Sox18*, are also expressed in the dorsal aorta of the mouse embryo and show similar effects in the formation of cell clusters with hematopoietic activity (Nobuhisa et al., 2014). Both *Sox17* and *Sox18* are significantly upregulated in hemogenic endothelial cells compared with endothelial cells isolated from the AGM at the time of the endothelial to hemogenic transition (Solaimani Kartalaei et al., 2015). Sox7 and Sox17 are not only required at this stage but also much earlier, between E7 and E8.5, as shown by single-cell gene expression measurements (Moignard et al., 2015). In that study, the authors showed that downregulation of Sox7 is key during erythroid development from the early mesoderm.

In addition to the stage-specific requirement for members of the SOX family, another hematopoietic transcription factor, C/EBPα, has also been implicated in the specification of developmental versus adult HSC types. Loss of *Cebpa* in *Mx1-Cre* adult transgenic mice confers a gain of fetal HSC-type characteristics on the bone marrow HSCs, including proliferation ratio and number of repopulating HSCs (Ye et al., 2013). Other factors, such as polycomb repressive complex 2 (PRC2), Bmi1 and Etv6 (Tel), function in adult HSCs but not in fetal liver HSCs (Xie et al., 2014; Copley et al., 2012). By contrast, *Prom1* was shown to be expressed by hemogenic precursor cells in the mid-gestation placenta (Pereira et al., 2016). Cells with a similar phenotype were also detected in the mid-gestation AGM but not among E13.5 fetal liver or adult bone marrow HSCs. This group further purified and analysed the Prom1^+^ hemogenic cells by mRNA sequencing, confirming both endothelial and (low-level) hematopoietic gene expression (Pereira et al., 2016). Other genes, such as *Fgd5* and *Ctnnal1* (also known as α-catulin), are expressed throughout HSC ontogeny. *Fgd5* is predominantly expressed in HSCs in both adult bone marrow and embryonic hematopoietic sites (Gazit et al., 2014). Although lethal at E12, *Fgd5* deficiency did not affect the generation and function of HSCs *in vivo*, suggesting a requirement for this factor outside of hematopoietic development. *Ctnnal1* was detected in both adult bone marrow HSCs and precursors of HSCs in the AGM (Acar et al., 2015; Zhou et al., 2016) and it too is not required for HSC development. Only 30% of the AGM pre-HSC gene signatures were shared by adult bone marrow HSCs (Zhou et al., 2016), suggesting that heterogeneity can be tissue- and developmentally derived. The fact that there are intrinsic molecular differences between HSCs at embryonic, fetal and adult stages, and that HSC heterogeneity already exists at the fetal liver stage of development raises questions as to when HSC subtypes first appear in development and how this might occur.

### The emergence of HSC subtype specification

Heterogeneity of HSCs may begin as early as the time when HSCs are first being generated, and involve a variety of inductive mechanisms and different microenvironments. In the mouse embryo, the first LT-HSCs are detected and generated at E10.5 in the AGM region. LT-HSCs are also found in other parts of the embryo, namely the yolk sac, vitelline/umbilical arteries, placenta and head. It might be that the generation of HSCs in these different sites at slightly different developmental stages is the source of – or at least a contributory factor to – the HSC lineage output heterogeneity found in the fetal liver. For example, embryonic head HSCs express Sca1GFP, a marker of all AGM HSCs (de Bruijn et al., 2002), and although Sca1GFP^+^ AGM cells undergo endothelial-to-hematopoietic transition (EHT) (Boisset et al., 2010), there is no evidence to suggest that EHT occurs in the embryonic head (Iizuka et al., 2016; Li et al., 2016). Thus, different mechanisms for HSC generation may exist. These, together with the diverse and dynamic developmental microenvironments might induce the generation of different HSC subtypes. Interestingly, a recent study showed that cultured mesenchymal stromal cell lines derived from different anatomical sites express distinct transcriptional programmes (Charbord et al., 2014). Since, *in situ*, the mesenchymal stromal cells form part of the HSC niche, it might be that their different transcriptional programmes reflect different molecular requirements for the maintenance of distinct HSC subtypes.

Hemogenic endothelial cells have been shown to be the direct precursors to HSCs, taking on a hematopoietic fate during a limited window of developmental time (Boisset et al., 2010; Chen et al., 2009; Zovein et al., 2008). Could these cells be the ultimate source of HSC heterogeneity? Hemogenic endothelial fate may be separated from cells with strictly endothelial fate at early stages, and is likely to be derived from the partitioning of mesodermal populations; for example, extraembryonic versus lateral versus axial fate. Differences in the hemogenic potential of ventral-lateral versus other mesoderm has been shown in chick embryonic aorta (Pardanaud et al., 1996). Human pluripotent stem cell hematopoietic differentiation cultures also show that hemogenic endothelium and vascular (arterial and venous) endothelium represent separate lineages (Ditadi et al., 2015). In mouse embryos, hemogenic endothelial cells are detected at least 2 days before HSCs are generated (Swiers et al., 2013), and there is evidence suggesting that presumptive aortic hemogenic endothelial cells are found in the lateral mesoderm of E7.5-E8.5 mouse embryos and require the transcription factors Etv2 and Hoxb6 (Kataoka et al., 2013). This hemogenic competence of endothelial progenitors was further shown to be restricted by *Runx1* silencing (Eliades et al., 2016). Interestingly, a subset of endothelial cells and hematopoietic cells located ventrally in the E10.5 AGM and a subset of endothelial cells and hematopoietic progenitor/stem cells in the E15.5 fetal liver can be derived from PDGFRα^+^ early paraxial mesodermal cells, as shown by cell tracing in which PDGFRα^+^ cells were labelled at E7.5-E8 by tamoxifen injections into pregnant females (Ding et al., 2013). However, PDGFRα is dispensable for the development of fetal liver hematopoiesis and *Pdgfra-*deleted mice die from a cephalic closure defect and skeletal abnormalities (Soriano, 1997). In addition, rare endothelial cells in the blood vessels of the head and cardiomyocytes were also marked. These results highlight the possibility that HSC subtypes might be determined at much earlier developmental stages than previously thought, and in different mesodermal populations.

Another possible source of HSC heterogeneity is the hematopoietic cells generated prior to the generation of LT-HSCs. In a neonatal transplantation scenario, the cKit^+^ CD34^+^ cell fraction of E9 yolk sac was shown to contribute to long-term chimerism, suggesting the existence of pre-HSCs (Yoder et al., 1997). Indeed, reaggregate cultures of AGM cells defined as pre-HSCs by phenotype and function were able to exhibit LT-HSC activity when transplanted into irradiated adult recipients (Taoudi et al., 2008). More recently, myeloid-lymphoid hematopoietic progenitors (also called immature HSCs or imHSCs) derived in the yolk sac and harboured in the E9.5 fetal liver were shown to contribute to definitive hematopoiesis when injected into irradiated adult recipients, albeit with low chimerism. Upon organ culture of E10 fetal liver in the presence of thrombopoietin, these cells were able to reconstitute the hematopoietic system of natural killer-competent mice (Kieusseian et al., 2012). More recent studies have shown that the number of pre-HSCs increases dramatically in the AGM from E9 and peaks at E11.5 (Rybtsov et al., 2016). This was further confirmed by single-cell RNAseq analysis showing that a subset of pre-HSCs in the AGM is enriched in cell division-related genes such as *Hmmr* (*Cd168*) (Zhou et al., 2016). The rapid decrease in pre-HSCs in the E11.5 AGM and abrupt, but similar, increase in the number of definitive HSCs in the fetal liver led the authors to suggest that subsets of definitive HSCs in the fetal liver originate from the maturation of AGM-derived pre-HSCs upon migration into their new microenvironment (Rybtsov et al., 2016).

Despite these interesting findings, there is still no direct *in vivo* evidence to demonstrate that distinct pre-HSCs or imHSCs contribute to HSC types found at later stages. Nonetheless, the asynchronous and graded cellular maturation could explain the heterogeneity of HSCs in the fetal liver. Individual cells might require different microenvironments to mature or may mature at different rates (Moignard et al., 2015). Based on these studies, it is now important to understand whether the pre-HSCs in the AGM, the imHSCs in the fetal liver and/or the HSCs in the yolk sac, placenta and head show distinct lineage outputs.

### Is more than one type of HSC generated in the AGM region?

HSC generation in the AGM region occurs in a polarised manner, with HSCs detected only on the ventral side of the aorta (Medvinsky et al., 2011; Taoudi et al., 2008). The initiation of HSC generation is tightly controlled by the local microenvironment: the ventral aortic endothelium and/or subaortic mesenchyme express molecules that trigger EHT and subsequent generation of HSCs (Richard et al., 2013; Durand et al., 2007; Pouget et al., 2014; Kaimakis et al., 2013; Robin and Durand, 2010). Of these molecules, BMP is expressed in the ventral aspect of the AGM in mesenchymal and aortic endothelial cells (Durand et al., 2007; Marshall et al., 2000) and has been shown to affect HSC activity in AGM explant cultures (Durand et al., 2007). The Runx1 and Gata2 transcription factors also show expression in the ventral aspect of the aorta at E10.5 and are required for hematopoietic progenitor and stem cell generation from hemogenic endothelium (Chen et al., 2009; Tober et al., 2013). Moreover, Notch signalling is essential for EHT *in vivo* (Gama-Norton et al., 2015), initiating endothelial cell fate change to a hematopoietic cell in the AGM through the Notch ligand jagged 1 (Robert-Moreno et al., 2008; Gerhardt et al., 2014; Guiu et al., 2014; Tang et al., 2013). Notch 1 is expressed in most of the cells of the intra-aortic hematopoietic clusters, similar to jagged 1 (Robert-Moreno et al., 2008). A signalling cascade linking some of the factors has been shown in zebrafish embryos. Here, BMP and Hedgehog act through VEGF/Notch signalling to polarise HSC emergence from the dorsal aorta (Gering and Patient, 2005; Wilkinson et al., 2009).

Two HSC subtypes that differ in activation by the BMP signalling pathway were identified in studies that examined HSC development in the AGM *in vivo* and *in vitro*. Based on a BMP activation marker, HSCs generated in the E11 AGM *in vivo* are of one type: BMP-activated (Crisan et al., 2015). However, when AGM explants are cultured prior to transplantation, two HSC subtypes are found: BMP-activated and non-BMP-activated HSCs (Crisan et al., 2016). Furthermore, it was found in AGM explant cultures that the non-BMP-activated HSCs were affected by the Hedgehog signalling pathway through VEGF, which is most likely produced by endothelial cells. Serial transplantation experiments suggested that whereas BMP-activated AGM HSCs yielded both BMP-activated and non-BMP-activated HSCs, non-BMP-activated HSCs yield only non-BMP-activated HSCs. This might indicate differences in the niche between the AGM and bone marrow. It has yet to be determined through lineage tracing whether the induction of all HSCs requires activation of the BMP signalling pathway.

The two HSC types that differ in their responsiveness to BMP were also found *in vivo* in the E12-E14 fetal liver. Limiting dilution transplantations of E14 fetal liver show that 80-90% of fetal liver HSCs are of the BMP-activated subtype (Crisan et al., 2015). Interestingly, the canonical BMP signalling pathway has been shown in *Smad1/5* knockout experiments to be dispensable for fetal liver HSC activity (Crisan et al., 2015; Singbrant et al., 2010), suggesting that, in the absence of BMP-activated HSCs, normal hematopoietic activity is maintained by the non-BMP-activated HSCs. When lineage output was assayed by clonal transplantation of fetal liver BMP-activated and non-BMP-activated HSCs, both fractions were found to contain balanced and lineage-biased HSC subtypes (Crisan et al., 2015). In contrast to the fetal liver, most bone marrow HSCs are of the non-BMP-activated type (Crisan et al., 2015). Clonal transplantations of bone marrow HSCs showed that all myeloid-lymphoid balanced HSCs are BMP-activated, whereas the majority of myeloid-biased HSCs are not activated by the canonical BMP pathway (Fig. 2).

Adult bone marrow HSCs are also controlled by TGFβ signalling (Yamazaki et al., 2009). Myeloid-biased and lymphoid-biased HSCs can be distinguished based on their response to the TGFβ signalling pathway, although not all HSC clones cleanly segregate between the two subgroups (Challen et al., 2010). Myeloid-biased HSCs are generally activated by TGFβ, whereas the lymphoid-biased HSCs are inhibited.

These results highlight the developmental changes that occur in HSCs and suggest that the bone marrow niche might influence HSC subtypes via the specific developmental growth factors that they secrete.

## Summary and future perspectives

HSC heterogeneity exists *in vivo* from early developmental stages, and different HSC subtypes can be detected as early as the fetal liver stage. HSC subtypes exhibit measurable functional properties, such as lineage output or self-renewal ability, and can be prospectively enriched based on surface expression levels of CD150 and cKit, or their responsiveness to the BMP or TGFβ developmental signalling pathways. Although as yet unknown, it is likely that gradients of these morphogens drive the emergence of different HSC types. The responsiveness to these signalling pathways is in association with, but not in complete correlation to, lineage output. Intrinsic molecular differences in HSCs from the fetal liver and adult stages have been found, and genes have been associated with specific HSC lineage outputs. However, the molecular networks that regulate the generation of specific HSC subtypes remain unknown, as do the developmental cellular precursors of HSC types. The changing balance and frequencies of HSC subtypes in the embryonic, fetal and adult microenvironments support the notion that distinct developmental niches are present and differentially affect the persistence and representation of specific HSC types and behaviours. Some studies are beginning to show that the maintenance of self-renewal of the HSCs that colonise the fetal liver depends on the interaction with the endothelial and perivascular cells (Iwasaki et al., 2010; Khan et al., 2016; Tamplin et al., 2015). Future studies should reveal the specific developmental niche compartments and factors that regulate the induction, maintenance, expansion and balance of HSC subtypes.

The physiological relevance of having different HSC types and subtypes is as yet uncertain. It is possible that lineage-biased HSCs in the bone marrow of the adult could confer an advantage during stress conditions or trauma in which the rapid replacement of a specific lineage of hematopoietic cells is required. In blood-associated cancers, some leukemias are of the myeloid type, whereas others are of the lymphoid type. Specific HSC subtypes in the adult or in the fetal stages might therefore serve as the targets of pre-leukemic events, and thus restrict the malignancy to the myeloid or lymphoid lineage. Additionally, the balance between the types of HSCs might change during hematological disease. Whether only HSCs are being mobilised under these conditions is unclear. A recent study proposed that steady-state hematopoiesis is derived from long-lived progenitors, both restricted and multipotent, rather than LT-HSCs (Sun et al., 2014). However, this model is not supported by hematological data on HSCs, erythroid progenitors and myeloid progenitors accumulated from aplastic anemia patients (Notta et al., 2016). Clonal tracking of genetically modified HSCs over 4 years following transplantation and hematopoietic reconstitution revealed that it takes ∼6-12 months to reach a normal and stable hematopoietic output. Importantly, this study demonstrated that steady-state hematopoiesis after transplant is maintained by both HSCs and multipotent progenitors (Biasco et al., 2016). Thus, it is important to properly investigate whether and how particular HSC subtypes contribute to specific hematologic disease. Our further understanding of the molecular regulators of specific HSC subtypes through transcriptome approaches should provide important information that can be applied in patient-specific treatments, particularly in leukemias to eradicate the affected HSC type while preserving the healthy HSC types for hematopoietic function.
