# Correction: The many faces of hematopoietic stem cell heterogeneity. Development doi: 10.1242/dev.114231

**DOI:** 10.1242/dev.160812

**Published:** 2017-11-15

**Authors:** Mihaela Crisan, Elaine Dzierzak

There was an error published in ‘The many faces of hematopoietic stem cell heterogeneity’ by Mihaela Crisan and Elaine Dzierzak (2016). Development **143**, 4571-4581 (doi: 10.1242/dev.114231).

Acknowledgement of funding from the European Research Council was inadvertently omitted. The revised Funding section appears below.

The authors apologise to readers for this mistake.

**Funding**

Work in our laboratories is funded by FES Netherlands Institute of Regenerative Medicine [101675]; the National Institutes of Health NIDDK [RO37 DK54077]; European Molecular Biology Organization [ALTF 260-2009]; ZonMW Dutch Medical Research Council [VENI 916-12-088]; Erasmus MC (Erasmus Medisch Centrum) [103.494]; a European Research Council Advanced Grant [341096] and a European Hematology Association Non-clinical Advanced Fellowship [2882492/R83480]. Deposited in PMC for release after 12 months.

